# The development of the larval nervous system, musculature and ciliary bands of *Pomatoceros lamarckii *(Annelida): heterochrony in polychaetes

**DOI:** 10.1186/1742-9994-3-16

**Published:** 2006-10-10

**Authors:** Carmel McDougall, Wei-Chung Chen, Sebastian M Shimeld, David EK Ferrier

**Affiliations:** 1 Department of Zoology, University of Oxford, South Parks Road, Oxford, UK

## Abstract

**Background:**

To understand the evolution of animals it is essential to have taxon sampling across a representative spread of the animal kingdom. With the recent rearrangement of most of the Bilateria into three major clades (Ecdysozoa, Lophotrochozoa and Deuterostomia) it has become clear that the Lophotrochozoa are relatively poorly represented in our knowledge of animal development, compared to the Ecdysozoa and Deuterostomia. We aim to contribute towards redressing this balance with data on the development of the muscular, nervous and ciliary systems of the annelid *Pomatoceros lamarckii *(Serpulidae). We compare our data with other lophotrochozoans.

**Results:**

*P. lamarckii *develops locomotory and feeding structures that enable it to become a swimming, planktotrophic larva within 24 hours. Formation of the trochophore includes development of a prototroch, metatroch and neurotroch, development of apical and posterior nervous elements at similar times, and development of musculature around the ciliary bands and digestive tract prior to development of any body wall muscles. The adult nervous and muscular systems are essentially preformed in the late larva. Interestingly, the muscular systems of the larvae and juvenile worms do not include the circular muscles of the body wall, which are considered to be plesiomorphic for annelids, although the possibility that circular muscles develop after these stages cannot be ruled out at this point.

**Conclusion:**

A comparison between polychaetes shows variability in the timing (heterochrony) of development of body wall muscles and elements of the nervous system. These heterochronies are one route for evolution of different life history strategies, such as adaptations to feeding requirements.

## Background

Analysis of molecular data has changed our understanding of bilaterian phylogeny, and produced the clades known as Ecdysozoa, Lophotrochozoa and Deuterostomia. With this new view of bilaterian relationships, it is apparent that established model organisms, such as *Drosophila melanogaster*, *Caenorhabditis elegans*, *Ciona intestinalis*, and *Mus musculus *are restricted to only two out of the three major phylogenetic groups, the Ecdysozoa and Deuterostomia [1]. Therefore, the accumulation of developmental and morphological data for taxa within the Lophotrochozoa is required to help distinguish patterns of conservation or convergence within the Bilateria and address questions about the evolution of the three major clades. Recently, some lophotrochozoan model systems have begun to be developed (for example, *Platynereis dumerilii *[2] and *Haliotis asinina *[3,4]), but studies on these organisms must be coupled with wider investigations in order to understand the diversity of developmental processes in the clade.

Within the Lophotrochozoa, many polychaete annelids display a classic vermiform and triploblastic body organisation [5] and possess relatively conserved gene sequences with respect to other bilaterians [6]. For these reasons they have been proposed by several authors to exhibit 'ancestral' bilaterian qualities and to be an excellent model system to study animal evolution and development [5,6]. However polychaete annelids are a speciose and morphologically diverse group, and investigation of their evolution and development needs to be coupled with further exploration of two areas. First, the phylogenetic relationships within the annelids are largely unresolved, with conflicting topologies achieved from molecular and cladistic methods [7-10]. Second, comparative embryological, developmental and genomic work should encompass a diverse range of annelids, as this will enable a more robust reconstruction of their ancestral condition.

The use of modern microscopy and immunohistochemical techniques has greatly aided our understanding of lesser known taxa, such as sipunculans [11], acoels [12], cycliophorans [13], entoprocts [14], and phoronids [15]. The most informative of these studies examine the development of various structures throughout the different life stages of the animal, enabling comparisons of the developmental pathways of different taxa and the formation of evolutionary hypotheses. While work of this nature has begun for the polychaete annelids [16-19], further work needs to be carried out to get a clear picture of development from embryo to adult in a variety of polychaete groups.

**Figure 1:**
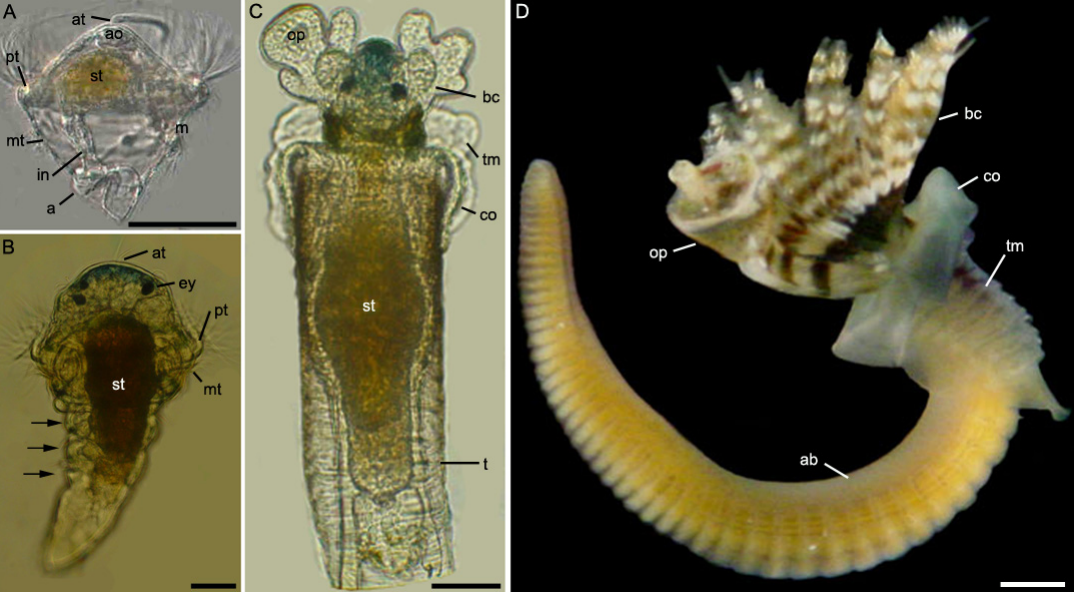
**Development of *P. lamarckii***. Light microscopical images. *Scale bars: *A-C = 50 μm; D = 1 mm. Apical is towards the top of the figure in all panels unless indicated. **A. **Complete trochophore, right lateral view. *a*, anus, *ao*. apical organ. *at*, apical tuft. *in*, intestine. *m*, mouth. *mt*, metatroch. *pt*, prototroch. *st*, stomach. **B. **Metatrochophore, dorsal view. *ey*, eyespot. Segments indicated by *arrows*. **C. **Early juvenile, dorsal view. *bc*, branchial crown. *co*, collar. *op*, operculum. *t*, tube. *tm*, thoracic membrane. **D. **Adult worm removed from tube, the anterior end possesses the branchial crown. *ab*, abdomen.

Segrove [20] described the development of the serpulid *Pomatoceros triqueter*, and the majority of his study (with the exception of his description of metamorphosed animals) has been corroborated by other work [21,22]. In addition, the literature contains some reconstructions of the serpulid nervous system by light microscopy [23-25]. This study aims to build on these previous studies by examining myogenesis, neurogenesis and ciliation in the tube-building polychaete, *Pomatoceros lamarckii *(Serpulidae) (see Fig. 1), using methods that are directly comparable to similar work being carried out on other invertebrate taxa. These events are investigated throughout embryogenesis, larval life and metamorphosis and the results represent a significant increase in the information available from the polychaete annelids.

## Results

### Development

**Figure 2:**
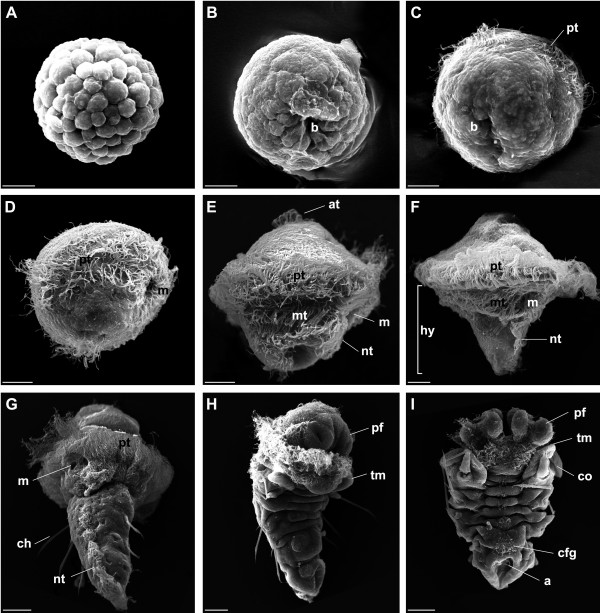
**SEM of the development of *P. lamarckii***. *Scale bars*: A-F = 10 μm; G-I = 20 μm. Apical is to the top of the figure in all panels. **A. **Blastula stage. **B. **Early gastrula, posterior view. The blastopore (*b*) is conspicuous at this stage. **C. **Posterior view of embryo showing closure of the lateral edges of the slit-like blastopore lip, resulting in formation of mouth and anus. Prototrochal (*pt*) cilia are visible at this stage. **D. **Early trochophore, right lateral view. Feeding cilia are visible around the mouth (*m*), which has reached its final location. The anus is located on the dorsal side of the animal (not shown). **E. **Complete trochophore, right lateral view. The apical tuft (*at*) is visible. The metatroch (*mt*) and neurotroch (*nt*) have formed. **F. **Late trochophore, right lateral view. Note elongation of hyposphere (*hy*) and general increase in size. **G. **Ventral posterior view of a metatrochophore larva with three segments, each marked by paired chaetae (*ch*). The neurotroch no longer reaches the mouth. **H. **Metamorphosing larvae, lateral view. The primary filaments (*pf*) of the branchial crown have begun to form, and loss of prototroch and metatroch has begun. The rudiments of the thoracic membrane (*tm*) are visible. **I. **Metamorphosed worm, dorsal view. The primary filaments of the branchial crown are prominent and the rudiments of the thoracic membrane and collar (*co*) are visible. The cilia of the newly formed faecal groove (*cfg*) are visible in a row along the dorsal midline. The anus (*a*) is now situated more terminally.

*P. lamarckii *embryos undergo equal spiral cleavage and form a hollow blastula (Fig. 2A) which, during gastrulation, forms a blastopore at the posterior pole (Fig. 2B). This blastopore elongates and becomes slit-like, the lateral edges of the slit draw together (Fig. 2C) and fuse to produce two apertures, which subsequently become the mouth and anus. In some annelid species these apertures may close and re-open later in development [26], but this was not observed in *P. lamarckii*. Within 24 hours the embryo has developed into a diamond-shaped, planktotrophic larva (Fig 2D). The mouth has migrated ventro-anteriorly and assumed its final position just below the ciliated prototroch, whereas the anus is situated posteriorly on the dorsal side. After the next 24 hours the development of the trochophore is complete. Additional ciliary bands include the metatroch and neurotroch, and an apical tuft is situated at the anterior extreme of the larva (Fig 1A, Fig. 2E). This 'complete larva' stage is maintained for several days before the hyposphere begins to elongate posteriorly (the 'elongation phase', Fig. 2F). The elongation phase is followed by the formation of a metatrochophore larva that possesses three segments, each with a set of chaetae (Fig. 1B, Fig. 2G). The metatrochophore larvae spend a period of time exploring surfaces before attaching and initiating metamorphosis, which involves the loss of ciliary bands, the emergence of adult structures such as the primary filaments of the branchial crown, the thoracic membrane, and collar, and the secretion of a mucous tube that later becomes calcified (Fig. 1C, D; Fig. 2H, I). *P. lamarckii *is known to require various cues in order to settle and metamorphose [27], and in several cases the biofilm that developed within the culture vessel was sufficient to induce settlement. Time taken to reach the metatrochophore stage varied from 9–16 days, even within a single culture separated into several dishes, possibly due to different larval densities or food levels. Due to this variation developmental stages are not assessed according to age but instead according to morphological features.

### Muscle development

**Figure 3:**
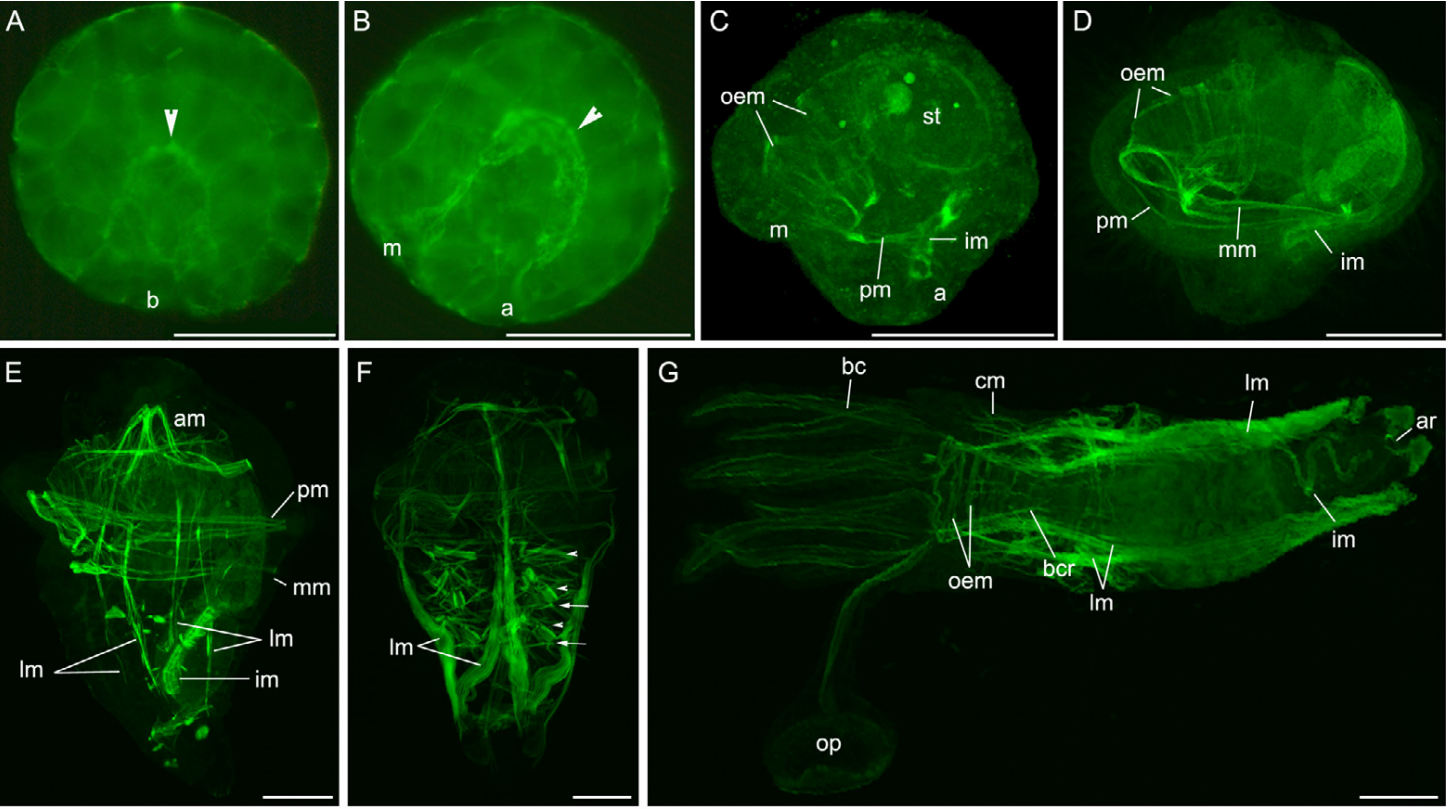
**Myogenesis in *P. lamarckii***. Visualised by phalloidin staining. *Scale bars *= 50 μm. Apical is towards the top in all panels unless indicated. **A**. Early gastrula, lateral view. A horseshoe-shaped strip of muscle can be seen along the invaginating gut (*arrowhead*). Blastopore (*b*) is present at the posterior of the embryo. Cell junctions (zonulae adhaerens) are reactive to phalloidin at this stage (and in B – out of focus in these images). **B**. Late gastrula/early neurula, left lateral view. Blastopore has split and mouth (*m*) migrates anteriorly leaving anus (*a*) at blastoporal pole. Developing gut muscle is indicated by arrowhead. In panels A and B there is faint staining along the cell membranes, presumably due to the phalloidin binding to the zonulae adhaerens cell junctions. **C**. Early trochophore, left lateral view. Oesophageal musculature (*oem*) has begun to form around the oesophagus and mouth. The prototroch muscle (*pm*) has formed and the intestinal muscle (*im*) runs along the intestine from stomach (*st*) to anus. **D**. Complete trochophore, left lateral view. Further development of features in C. A metatroch muscle (*mm*) has also developed. **E**. Elongating trochophore, left lateral view. Longitudinal muscles (*lm*) run in pairs ventro-laterally and dorso-laterally towards the posterior of the larva. These fibres converge apically in the anterior muscle mass (*am*). **F**. Metatrochophore, latero-ventral view. Longitudinal muscles are more prominent and chaetal muscles have developed corresponding to the three new body segments (*arrowheads*). A thin, transverse muscle is seen in each segment (*arrows*, anterior most muscle obscured in this image). **G**. Juvenile, dorsal view. Anterior to the left. Longitudinal, oesophageal and intestinal muscles are still present. Muscles can be seen running along each filament in the branchial crown (*bc*) and the operculum (*op*). V-shaped branchial crown retractors (*bcr*) are present in the pharyngeal region. Small muscles can be seen in the newly developed collar (*cm*) and a ring of muscle is visible around the anus (*ar*).

Muscle development was visualised using phalloidin, a compound that binds to F-actin. Staining is first visible just after gastrulation has commenced, as a horseshoe-shaped strip associated with the developing gut (Fig. 3A). As the mouth migrates anteriorly away from the anus, F-actin staining in the oesophagus, stomach and intestine becomes more obvious (Fig. 3B). The zonulae adhaerens (a particular type of cellular junction) also bind phalloidin at this stage (Fig. 3A, B).

At the early trochophore stage (Fig. 3C) bands of circular muscle have begun to form around the oesophagus and a large muscle can be seen running alongside the intestine from its origin near the stomach to the dorsally located anus. The prototroch muscle ring has also developed by this stage. Once a complete trochophore has developed, the oesophageal musculature is more prominent, particularly around the mouth. An additional circular muscle ring has developed and is associated with the metatroch (Fig. 3D).

The elongation phase of the larva (Fig. 3E) is associated with the development of many more muscles, most of which are retained after metamorphosis. At this stage the four longitudinal muscles develop, which all originate at a similar position anterior to the stomach and extend posteriorly in two pairs, one ventro-laterally and the other dorso-laterally. The origin of these muscles is within the anterior muscle mass, which consists of several loops of fibres that either wrap around the stomach dorsally or arch anteriorly to come into close contact with the apical organ before progressing ventrally to connect with the oesophageal musculature. At the posterior end of the animal another muscle mass is present, running obliquely from the ventral surface (anterior to the anal vesicle) to the dorsal surface near the anus. The prototroch muscle ring now consists of two distinct bands, whereas the metatroch muscle ring consists of one band that follows the metatroch around its entire circumference. An extra muscle band can be seen in close proximity of this band on the dorsal side of the animal, however this muscle band passes anterior to the mouth on the ventral side.

The metatrochophore (Fig. 3F) exhibits an increase in muscle fibres within the four longitudinal muscles, making them the most prominent muscles in the animal. The number of muscle fibres in the prototroch and metatroch also increases (data not shown). Complex musculature associated with the formation of chaetae is present in three pairs corresponding with the three larval segments. A thin transverse muscle is also present in each segment and appears to associate with the chaetae.

After metamorphosis the majority of the metatrochophore musculature is still present (Fig. 3G). The four longitudinal muscles run the length of the animal, inserting at the base of the branchial crown. The intestinal muscle traces the majority of the intestine. The oesophageal musculature is funnel shaped and its position reflects the migration of the mouth which is now present at the anterior of the organism. New musculature includes the opercular and branchial crown muscles, the branchial crown retractors, the collar muscles and a ring of muscle around the anus. The prototroch and metatroch circular muscles are apparently resorbed along with their associated ciliary bands.

### Ciliary bands

**Figure 4:**
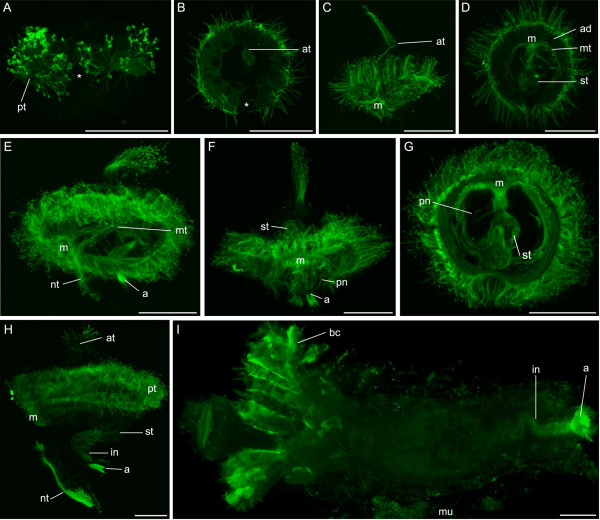
**Ciliation in *P. lamarckii***. Visualized by immunohistochemistry with anti-acetylated tubulin. *Scale bars *= 50 μm. **A**. Early gastrula, dorsal view, apical is to the top. The cilia of the prototroch (*pt*) are present and function at a very early stage. The ring is initially open on the dorsal side (*). **B**. Gastrula, apical view. Shortly after prototroch formation the apical tuft (*at*) is formed. The prototroch is still open dorsally. **C**. Early trochophore, ventral view, apical is to the top. The mouth (*m*) has now reached its final location. **D**. Early trochophore, posterior view. The adoral ciliary zone (*ad*) has begun to form in a ring lateral to the mouth. Posterior to this the metatroch (*mt*) begins to form. Ciliation in the oesophagus and stomach (*st*) is evident at this stage. **E**. Complete trochophore, left lateral view, apical is to the top. At this stage ciliary bands including the longitudinal neurotroch (*nt*) are complete. A tuft of cilia emerges from the anus (*a*). **F**. Complete trochophore, ventral view, apical is to the top. Paired protonephridia (*pn*) run anteriorly from the anus. **G**. Complete trochophore, posterior view. Extensive ciliation in the oesophagus and stomach is visible. **H**. Metatrochophore, left lateral view, apical is to the top. Ciliation is similar to previous larval stages, but protonephridia are not visible. The neurotroch no longer reaches the mouth. **I**. Juvenile, ventral view, anterior to the left. The juvenile shows reduced ciliation, with the branchial crown (*bc*), intestine (*in*) and anal tuft (*a*) the only features visible. Remnants of the mucous tube (*mu*) are apparent.

The first ciliated structure to develop is the prototroch, which forms primarily from two rows of cells soon after gastrulation (Fig. 2C; Fig. 4A). Each cell is multiciliate, and initially there is a break in the prototroch on the dorsal side of the animal. Before this break is closed, the cilia of the apical tuft are formed (Fig. 4B). At these stages the ciliated cells themselves are reactive to anti-acetylated tubulin (Fig. 4A, B).

As the larvae near the feeding stage (Fig. 2D; Fig. 4C, D) the mouth opening has migrated to its ventral position beneath the prototroch. Underneath the prototroch a broad band of shorter cilia (the adoral ciliary band) forms. A third band of cilia, the metatroch, also forms at this time and encircles the larva at the level of the posterior edge of the mouth. Cilia in the oesophagus and stomach are also obvious. The ciliated bands, including the neurotroch that runs longitudinally along the ventral side of the larva posterior to the mouth, are fully developed in the complete trochophore (Fig. 2E; Fig. 4E, F, G). There is no obvious ventral break in the metatroch to allow the neurotroch to reach the mouth. The anus is clearly distinguishable by the tuft of cilia that emerge from it, and paired protonephridia can be traced from the anus to a region in the proximity of the metatroch (Fig. 4F, G).

In the elongating larva and metatrochophore ciliary patterns remain much the same, except that the neurotroch becomes denser towards the posterior pole of the animal and no longer reaches anteriorly to the mouth (Fig. 2G; Fig. 4H). Much of the larval ciliation is lost at metamorphosis, with the exception of that in the gut. Cilia form on the developing branchial crown (Fig. 4I), and in later stages bands of cilia, which are the beginnings of the adult faecal groove, appear on the dorsal side of the animal (Fig. 2I).

### FMRFamidergic nervous system

**Figure 5:**
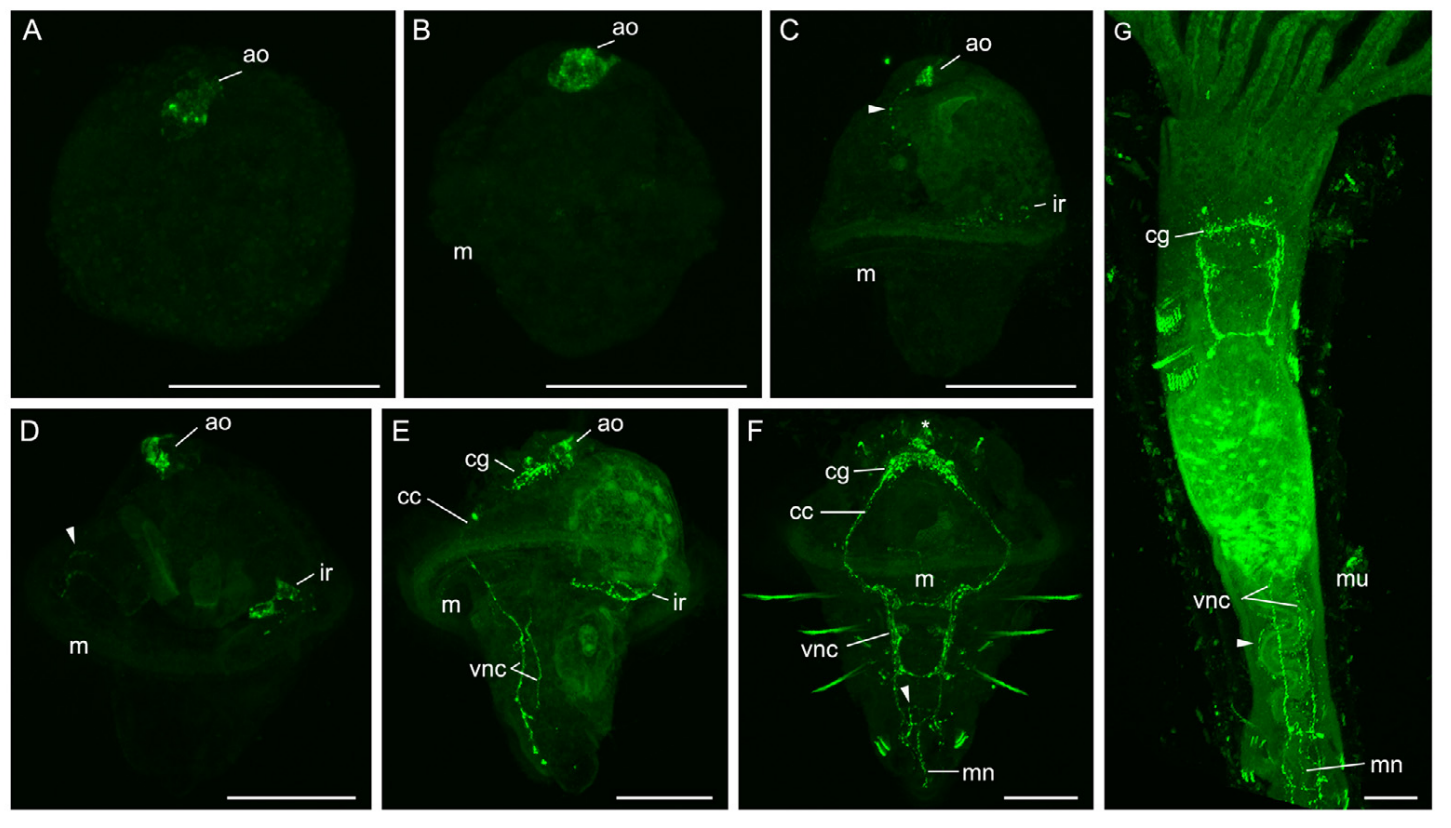
**FMRFamidergic nervous system development in *P. lamarckii***. *Scale bars *= 50 μm. Apical is to the top of the figure in each panel unless indicated. **A**. Early gastrula, lateral view. A FMRFamidergic region can be seen in the apical organ (*ao*). **B**. Early trochophore, left lateral view. The mouth (*m*) has reached its final location, but no further FMRFamidergic structures are visible. **C**. Early trochophore, left lateral view. A FMRFamidergic nerve ring is visible around the opening of the stomach to the intestine (*ir*), and a ventral process can be seen leading posteriorly from the apical organ (*arrowhead*). **D**. Late trochophore, left lateral view. A nerve ring around the mouth is visible (*arrowhead*). **E**. Elongating trochophore, left lateral view. Elements of the cerebral ganglion (*cg*) are now developing, and are connected to the ventral nerve cords (*vnc*) by the circumoesophageal connectives (*cc*). **F**. Metatrochophore, ventral view. The cerebral ganglion is now fully formed and additional FMRFamidergic fibres are seen lateral to it. Elements of the apical organ are still visible (*). Commissures are apparent between the ventral nerve cords. A medial nerve (*mn*) runs between the ventral nerve cords and is more evident at the posterior pole of the larva. Fibres can be seen encircling the intestine (*arrowhead*). Chaetae and uncini are autofluorescent. **G**. Juvenile, ventral view, anterior is towards the top. The FMRFamidergic nervous system is similar to that in the metatrochophore. Fibres can be seen along each side of the intestine (*arrowhead*). Remnants of the mucous tube (*mu*) are apparent.

The first FMRFamidergic signal appears in the apical organ just after the appearance of the apical tuft (Fig. 5A). Staining in this region becomes more intense during early development (Fig. 5B) and is restricted to the apical organ until the trochophore is almost fully developed. At this stage (Fig. 5C), FMRFamidergic processes with a ventral trajectory can be seen emerging from the apical organ. A nerve ring is also visible around the opening from the stomach into the intestine. Another much more weakly fluorescent nerve ring can be seen in the oesophagus near its entry into the stomach (data not shown). This nerve ring is much more visible in the complete trochophore (Fig. 5D), where it is much larger in diameter and in closer proximity to the mouth.

In the elongating larva FMRFamidergic fibres appear in the paired ventral nerve cords (Fig. 5E). These connect to the cerebral ganglion, which develops in pockets lateral and posterior to the apical organ, via the circumoesophageal connectives. The ventral nerve cords also associate with the nerve ring that encircles the mouth. Commissures begin to form between the ventral nerve cords. In the metatrochophore (Fig. 5F) the commissures and associated immunoreactive cells are visible and correspond to the newly formed segments. The cerebral ganglion has formed, and the apical organ is reduced but still present. Paired FMRFamidergic fibres can be seen on either side of the cerebral ganglion and may be associated with the eyespots. Fainter fibres can be seen encircling the mouth, and, more posteriorly, the lower intestine. A faint nerve fibre (medial nerve) can be seen between the two ventral nerve cords, this becomes more pronounced at the posterior extremity of the larva.

After metamorphosis the apical organ is no longer visible, the most anterior FMRFamidergic structure is now the cerebral ganglion (Fig. 5G). As in the larvae, this cerebral ganglion is connected to the two ventral nerve cords and associated commissures that continue to the posterior of the animal (obscured partly in the figure by gut autofluorescence). The medial nerve is still present. In addition to these retained features, fibres can be seen running alongside the intestine.

### Serotonergic nervous system

**Figure 6:**
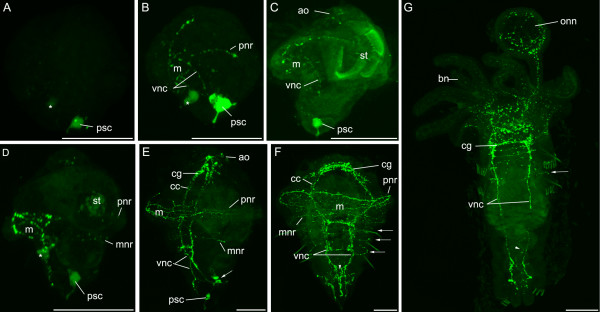
**Serotonergic nervous system development in *P. lamarckii***. *Scale bars *= 50 μm. Apical is to the top of the figure in each panel unless indicated. **A**. Gastrula, left lateral view. One posterior cell (*psc*) is highly reactive for anti-serotonin. Another cell in the vicinity of the mouth is also reactive (*). Very faint, scattered fluorescent signal marks the beginning of formation of nerve fibres. **B**. Early trochophore, left lateral view. The posterior serotonergic cell sends processes apically. These fibres are the rudiments of the ventral nerve cords (*vnc*) and associate with processes that innervate the prototroch nerve ring (*pnr*). Fibres encircle the mouth (*m*). The second serotonergic cell (*) now lies posterior to the mouth and is associated with the suboesophageal ganglion.**C**. Early trochophore, left lateral view. The prototroch nerve ring is complete and runs behind the stomach (*st*). The neuropil has begun to appear in the apical organ (*ao*). Some gut autofluorescence is visible. **D**. Complete trochophore, left lateral view. A metatroch nerve ring appears (*mnr*). Some gut autofluorescence is visible. **E**. Elongating trochophore, left lateral view. The cerebral ganglion (*cg*) is developing and is connected to the ventral nerve cords by the circumoesophageal connectives (*cc*). The prototroch nerve ring now consists of multiple fibres. Serotonergic cells near the anus are connected to the ventral nerve cords by small fibres (*arrow*). **F**. Metatrochophore, ventral view. The paired ventral nerve cords gain commissures and converge posteriorly (*arrowhead*). Circular nerves can be seen in the periphery of each segment (*arrows*). Chaetae are autofluorescent. **G**. Juvenile, ventral view, anterior is to the top. The branchial nerves (*bn*) and opercular nerve net (*onn*) are serotonergic. A serotonergic fibre can be seen running along the intestine (*arrowhead*). Chaetae and uncini are autofluorescent.

The first serotonergic cell appears at the blastoporal pole of the embryo at approximately the same stage that FMRFamide signal is visible in the apical region (Fig. 6A). This cell has a large process that it sends to the very posterior of the larva; the cell and its process persist throughout larval development. A second, less reactive cell becomes visible shortly after the posterior serotonergic cell and is situated in the mouth region.

When the mouth has reached its final ventral position under the prototroch, much of the trochophore nervous system is in place (Fig. 6B, C). The prototroch possesses a serotonergic nerve ring, and there are several fibres associating with the mouth and oesophagus. A process that runs midventrally to the apical organ also originates at the junction between the oesophageal nerves and the prototroch nerves. The posterior serotonergic cell sends processes anteriorly; these connect with the prototroch and are the precursors of the ventral nerve cords. The second, more weakly fluorescent posterior cell is now located below the mouth in the position of the future suboesophageal ganglion. A nerve ring later appears that runs around the larva in the vicinity of the metatroch (Fig. 6D).

At the elongation stage (Fig. 6E), the serotonin reactivity in the apical organ increases and high levels of serotonin are present in the developing cerebral ganglion and circumoesophageal connectives. The cerebral ganglion, apical organ, circumoesophageal connectives and ventral nerve cords are reactive for both anti-FMRFamide and anti-serotonin, although it is not known if these co-localise in the same cells or fibres. Paired serotonergic fibres innervate the prototroch and there is an increased complexity in the proximity of the mouth where these fibres appear to branch into the oesophagus. Cell bodies near the anus are connected to the ventral nerve cords by small serotonergic fibres. Commissures have begun to form between the ventral nerve cords, with the anterior-most commissure appearing first.

The apical organ is not visible by serotonin reactivity at the metatrochophore stage (Fig. 6F). The ventral nerve cords consist of multiple fibres, and commissures are present between the cords in a segmentally repeated pattern. The prototroch and metatroch still possess a serotonergic nerve ring, and additional serotonergic rings can be seen around the animal in each segment. At the posterior of the animal, the two nerve cords appear to converge at the midline and form three processes at the tip. Dorsally, a serotonergic meshwork is associated with the intestine. The posterior cell and its process are still visible (obscured by the ventral nerve cords in Fig. 6F).

In the juvenile (Fig. 6G), extensive serotonergic innervation of the branchial crown and operculum is evident. The cerebral ganglion, ventral nerve cords and associated commissures are reactive. Circular bands are visible in some segments and one fibre runs medially along the gut. Overall, the juvenile anti-serotonin and anti-FMRFamide patterns in the central nervous system are very similar.

## Discussion

### Myogenesis

Annelid muscles have been studied in depth in the adults of several species [8,28,29] but investigations of the ontogeny of these muscle systems are largely lacking. Phalloidin labelling of F-actin has facilitated studies of myogenesis in many invertebrate phyla, including acoels [12], molluscs [30,31], entoprocts [14], phoronids [15], cycliophorans [13] and sipunculans [11]. One study of phalloidin labelling in *Capitella sp. 1 *larvae [16] shows that myogenesis in this polychaete annelid first begins with a ring of muscle around the stomodaeum and is followed by the development of numerous longitudinal and then circular body-wall muscles. These muscles persist in the adult. *Capitella*, however, possesses non-feeding larvae that are brooded and emerge as highly derived metatrochophores, skipping the typical trochophore stage [16,32]. In *P. lamarckii*, myogenesis is first seen in the developing gut. Oesophageal muscles and the prototroch and metatroch muscle bands form in the trochophore larva, and body wall musculature is not added until the trochophore elongates to form the metatrochophore. It therefore appears that a change in timing has occurred in the development of *P. lamarckii *and *Capitella*, with *Capitella *developing body wall musculature first, and *P. lamarckii *forming the musculature of the digestive system before any body wall muscles. This is an example of heterochrony in development and appears to correlate with the different feeding strategies of the two species, with the planktotrophic *P. lamarckii *developing feeding musculature earlier in development whereas in the non-feeding *Capitella *larvae the process is delayed. Which, if either, represents the ancestral pattern of myogenesis is a moot point, as it is a matter of debate as to whether lecithotrophic or planktotrophic larvae are basal [33-35]. In both cases, however, the body wall musculature that develops in the larva persists through to adulthood. This is likely to be a common feature of polychaete annelid larval development.

A prototroch muscle ring has been observed in many molluscan larvae, including polyplacophora, bivalves and gastropods, but is distinctly lacking in the scaphopod *Antalis entalis *[30,31]. In addition, the sipunculan *Phascolion strombus *lacks a muscle ring associated with its prototroch [11]. Although the homology of the mollusc and annelid prototroch (and its muscle) is likely, based on embryological considerations [26], the possible (although still uncertain) placement of the Sipuncula as a sister group to the annelids [36,37] raises the possibility that the prototroch muscles are independent innovations in molluscs and annelids. A more likely explanation is that the sipunculan prototroch and its associated structures are secondarily reduced. In sipunculans, the prototroch does not develop to its full extent, and the animal uses a post-oral ring of cilia for locomotion [38]. This could explain the lack of muscles (and nerves) in the sipunculan prototroch, and does not invalidate the hypothesis that the prototroch is a homologous structure in trochozoans.

In addition to the prototroch muscle ring, *P. lamarckii *also possesses a metatroch muscle ring. This muscle ring has been described in other polychaete species [19,24]; however its distribution outside the Annelida is undocumented.

*P. lamarckii *exhibits no circular muscle in the body wall (except for the prototroch and metatroch muscle bands) in larvae or juveniles. Muscles repeated in the segments of later stages are components of the chaetal musculature (see Fig. 3F), and should not be confused with circular muscles [28]. The widely accepted view on adult annelid musculature is that the body wall consists of an outer layer of circular muscle and an inner layer of longitudinal muscle [5]. This general assumption has been challenged by the finding that members of several groups of polychaete annelids lack circular muscle altogether [28,29]. The transverse muscles seen in *P. lamarckii *are not likely to be rudiments of these circular muscles as they are associated with the chaetae and not with the body wall. Thus reinvestigation into the ancestral state for body wall muscles in the Annelida is warranted, which must be combined with a more robust annelid phylogeny [10,28,29].

### Ciliation

*P. lamarckii *larvae possess a conspicuous prototroch, which is the first ciliary feature to appear on the developing embryo. The prototroch ring is initially open on the dorsal side of the larva, as has been documented in other polychaetes [18,26], and appears to form from two rows of cells. Additional cells may contribute to the prototroch as seen in other annelids [39], but these were not visible here. Later ciliation in *P. lamarckii *is quite typical for trochophore larvae and consists of an apical tuft, adoral ciliary zone, metatroch, and neurotroch, i.e., all the components of the 'opposed-band feeding' system [34]. A ventral break in the metatroch, allowing the neurotroch to reach anteriorly to the mouth, has been described previously for some annelids [24,26]. This does not appear to be the case in *P. lamarckii*, instead the metatroch appears to be continuous with cilia that run inside the oesophagus. *P. lamarckii *lacks a telotroch at all stages of development. Early in development, cilia become visible in the developing gut and tubulin staining clearly highlights a pair of protonephridia, which either lead to the anus or a paired opening in the vicinity of the anus. These protonephridia can not be seen in later stages, and possibly degenerate, as is the case in some phyllodocid larvae [40]. Anti-tubulin immunohistochemistry has been used in numerous studies to visualise axon fibres in developing larvae (for example see [18,41,42]), however in *P. lamarckii *intense staining of ciliated bands made visualisation of neural elements impossible until the late stages of development.

### Neurogenesis in *P. lamarckii*

Immunohistochemical analyses of neurogenesis have been carried out on the larvae of three polychaetes, *Polygordius lacteus *(Polygordiidae) [19], *Phyllodoce maculata *(Phyllodocidae) [18] and *Pomatoceros lamarckii *(Serpulidae) (this study). These larvae show basic similarities; all develop paired ventral nerve cords, possess a suboesophageal ganglion, an apical organ and later a cerebral ganglion, develop a prototrochal nerve ring and a complex of nerves in the oesophagus (described in detail in [24]). The larvae differ in terms of the timing of formation of various features, and there are some differences in neuronal structures. Each of these larvae are morphologically and developmentally different, with *P. lamarckii *possessing a free-swimming planktotrophic larva, *P. maculata *possessing an initially encapsulated larva [18] and *P. lacteus *possessing a free-swimming larva that develops adult segments inside the hyposphere in later larval stages [19,43]. *P. maculata *and *P. lamarckii *are most likely phylogenetically distant from one another, while the position of the Polygordiidae is still unresolved [43]. Neurogenesis in a species of Myzostomidae has also been described but will not be discussed here as the phylogenetic placement of this species is uncertain and their body plans are highly derived, due to their parasitic lifestyle [17,42].

The finding that neurogenesis in gastropods proceeds from the posterior periphery of the organism to the anterior and centre challenged the widely held view that neurogenesis follows a strictly anterior to posterior pattern in these animals [44,45]. In response to this Voronezhskaya and colleagues [18] examined neural development in the polychaete *Phyllodoce maculata *to investigate whether this posterior-first pattern was more widespread. In *P. maculata *the first immunoreactive cell is a posterior serotonergic cell that gives rise to the ventral nerve cords. Immunostaining in the apical region is not seen until twelve hours later. Therefore, neurogenesis in *P. maculata *is similar to that seen in molluscs, i.e. progressing from the periphery to the centre.

The posterior serotonergic cell seen first in *P. maculata *neurogenesis is strikingly similar to the posterior serotonergic cell seen early in *P. lamarckii *development. Both cells are in a similar position in the embryo and contribute fibres to the ventral nerve cords. In *P. lamarckii*, however, this cell arises at a very similar time to (if not after) the first anti-FMRFamide signals in the apical organ. It is unknown whether neurogenesis in *P. lacteus *begins at the anterior or posterior of the embryo because data on the early stages is not available [19]. In light of these studies, peripheral to central neurogenesis does not appear to be the case in all polychaetes. We suggest the mode of neurogenesis is variable and subject to heterochronic shifts depending on the life history of the organism, i.e., that planktotrophic species require the development of anterior and posterior neural elements early in development for coordination of swimming and/or feeding, whereas lecithotrophic species do not require the anterior elements until later in development.

The neuroanatomy of the polychaetes studied showed several features that are shared between many trochozoans. A prototroch nerve ring is found in all three species, and a metatroch nerve ring is found in *P. lamarckii *and *P. lacteus *(*P. maculata *lacks a metatroch). In both *P. lacteus *and *P. maculata *these nerve rings are reactive for both serotonin and FMRFamide, whereas in *P. lamarckii *only serotonin staining is observed. These nerve rings possess multiple fibres, an observation that has also been made in the prototroch of bivalve molluscs [46], and the marginal ciliary band of the pilidium larvae of nemerteans [47], while polyplacophorans and ectoprocts have a more extensive nerve net [48,49]. Therefore, complex innervation of ciliary bands may be a common feature of Lophotrochozoa. A medial nerve, situated between the ventral nerve cords, is only seen in *P. lamarckii*, although it appears to develop in the very late larvae and may have been present in stages outside the scope of the study by Voronezhskaya et al. (2002). This median nerve is likely to be homologous to the 'neurotroch nerve' of *Spirobranchus polycerus *described by Lacalli [24] using transmission electron microscopy. Both the prototroch nerve ring and the median nerve have been identified in several other trochozoan taxa, although the neurotransmitter content of these structures varies [11,38,45,50]. Neurotransmitter content of a neuron can change during development [51,52], therefore the difference in neurotransmitters does not necessarily mean these structures are not homologous. This also applies to the ventral nerve cords, which are considered to be homologous in protostomes but also differ in neurotransmitter content [53].

Comparisons with earlier reconstructions of the serpulid nervous system using light microscopy [24] shows that visualisation using antibodies against FMRF-amide and serotonin gives a comprehensive picture of neurogenesis in these animals. From his reconstructions, Lacalli [24] stated that the larval nervous system in *S. polycerus *consisted of two separate parts: the pre-trochal part comprising the apical organ, prototroch, and connections between, and the oral part comprising pharyngeal nerves, the metatroch nerves and the neurotroch nerve. He also proposed that the adult nervous system develops entirely separately from the larval nervous system. This latter finding was disputed by Voronezhskaya and colleagues [18], and is not supported by this study. Both Voronezhskaya et al. [15] and ourselves were able to detect the posterior serotonergic cell and the rudiments of the ventral nerve cords, which were either missed in the *S. polycerus *reconstructions or are not present in this particular polychaete annelid [24]. In the immunohistochemical studies on *P. maculata *connections were seen between the oesophageal nerves and the prototroch [18]. In our *P. lamarckii *study connections between what Lacalli [24] calls the pre-trochal and oral systems were also detected, notably between the metatroch and ventral nerve cords. The larval nervous system of polychaetes is thus interconnected, and reorganises into the adult nervous system at metamorphosis.

In the metatrochophore of *P. lamarckii*, the serotonergic ventral nerve cords have a double appearance (Fig. 6F). The pattern seen here strongly resembles that seen in Lacalli's reconstruction of *S. polycerus *[24], where the inner cords give rise to the anterior-most commissures and the outer cords give rise to those toward the posterior pole. However, it is not known how integrated the two parts of the cords are to each other, and therefore whether they should be treated as separate nerve cords.

### Phylogenetic considerations

Molecular phylogenetic analyses have shown that Annelida and Sipuncula are closely allied taxa [54,55]. Indeed, the nervous systems of the two groups are similar [11]. The ventral nerve cords and cerebral ganglia are reactive for serotonin and FMRFamide in both taxa, and the dominant neurotransmitter in the apical organ in early stages is FMRFamide. Both *P. lamarckii *and the sipunculan *Phascolion strombus *possess an FMRFamidergic medial nerve and peripheral nerves associated with the viscera. In late larval stages convergence of serotonergic fibres in the ventral nerve cords can be seen posteriorly in both species. Obviously, these features are not shared by all groups of annelids and sipunculans, for example the annelids *Phyllodoce maculata *and *Polygordius lacteus *do not show any evidence of a medial nerve [18,19]. However the fact that members of both phyla possess these characters is consistent with a close relationship between the two taxa. Similar comparisons with other lophotrochozoan taxa can be made when more data are available.

## Conclusion

This study adds to the growing bank of morphological and developmental data that is accumulating for lophotrochozoan taxa. This data and comparisons between groups will help us to answer major questions about the evolution of the Bilateria. For example, this study is consistent with previous hypotheses that the prototroch nerve ring, apical organ, ventral nerve cords and medial nerve are basal characters for trochozoans [49,53]. It also shows that the larval nervous system is interconnected rather than consisting of unconnected pre-trochal and oral systems, and is rearranged at metamorphosis to form the adult nervous system, observations that have bearings on the evolution of the biphasic life cycle.

The comparison of the results from this study with those of other polychaetes gives major insight into the diversification of annelid body plans. It highlights that the timing of developmental events such as anterior and posterior nervous system formation and gut and body wall myogenesis is variable between polychaete taxa. Heterochronies, such as changes in the timing of developmental events or switching of two processes in developmental time, can cause major changes in the gross morphology of animals (see [56] for review). These changes enable animals to develop different life history strategies, such as early feeding. Heterochrony can thus be a major adaptive route in evolution and further investigation into its molecular basis will reveal the mechanisms by which this evolution occurs.

## Methods

### Animal collection and larval culture

Adult *P. lamarckii *(Quatrefages, 1865) were collected from the littoral zone at Tinside, Plymouth, UK, and were maintained at 12°C in a recirculating aquarium system. Adult worms (Fig. 1D) were removed from tubes by breaking open the posterior section and forcing the animals backwards using a blunt probe. Fertile animals began releasing gametes at this stage. Male and female worms were immediately transferred to separate Petri dishes or six-well plates containing filtered, UV treated seawater (FSW). Eggs were rinsed through a 100 μm cell strainer (Falcon) to remove tube debris, and then collected in a 40 μm cell strainer (Falcon). The strainer was then transferred to a Petri dish containing a dilute sperm suspension, agitated, and left for 10 minutes to allow fertilisation to occur. Eggs were rinsed and gently washed into a 300 ml Pyrex crystallisation dish containing 250 ml FSW. These dishes were covered and incubated at 19°C. From 24 hours post fertilisation (hpf) onwards larvae were fed on the red alga *Rhinomonas reticulata*.

### Fixation

Fixation and immunolabelling followed the methods of Wanninger and colleagues [11]. Larvae were fixed in 4% paraformaldehyde in 0.1 M phosphate buffered saline (PBS) at 4°C overnight, washed three times in PBS and then stored at 4°C. Metatrochophore larvae and juveniles were relaxed in 7% MgCl_2 _in FSW prior to fixation.

### Immunolabelling

Calcareous tubes of juveniles were dissolved in 0.1 M EGTA for approximately 15 minutes at room temperature (RT), which was followed by four washes in PBS. Permeabilisation of tissue was performed in 0.1 M PBS, 0.1% Triton X-100 (PBT) for 15 minutes at RT, and was followed by blocking of non-specific binding sites overnight at 4°C in PBT with 2% bovine serum albumen (block-PBT). The following steps were all performed at 4°C. Animals were incubated in 1:1000 (monoclonal anti-acetylated tubulin, Sigma), 1:40000 (polyclonal serotonin 5-HT antiserum, Immunostar), or 1:200000 (polyclonal FMRF-Amide antiserum, Immunostar) dilutions of the primary antibodies in block-PBT for 24h. After four 1h washes in block-PBT specimens were incubated in 1:800 dilutions of Alexa fluor 488 goat anti-rabbit IgG (serotonin and FMRF-amide) or Alexa fluor 488 goat anti-mouse IgG (tubulin) (Molecular Probes) in block-PBT for 24h. Specimens were then washed four times in PBS for over 1h, immersed in 60% glycerol in PBS and mounted in Vectashield with DAPI (Vector Labs). Negative controls were obtained by omitting primary antibodies and showed no specific staining.

For F-actin staining all steps were performed at RT. Specimens were permeabilised in PBT for 1h. A 1:40 dilution of Oregon Green 488 phalloidin (Molecular Probes) in PBT was added to animals for 1h and was followed by three 15 minutes washes in PBS. Specimens were then immersed in 60% glycerol in PBS and mounted in Vectashield with DAPI. Negative controls were obtained by omitting phalloidin and showed no specific staining.

A minimum of 40 individuals of each larval stage (or 20 juveniles) were investigated per staining procedure. Fluorescence microscopy was performed on a Zeiss Axioskop 2 and images were captured using Axiovision 4. Several representative individuals from each developmental stage were studied further using confocal microscopy, which was performed on a Leica DMIRE2 microscope equipped with a Leica TCS SP2 confocal unit.

### Scanning electron microscopy

Embryos/larvae were fixed in a mixture of 75 mM sodium cacodylate, 3% glutaraldehyde and 1% of calcium chloride for at least 2h. The fixative was replaced by two 5 minutes exchanges of 0.1 M sodium cacodylate and then 1% osmium tetroxide in 0.1 M sodium cacodylate for 1h. After the specimens were rinsed twice with 0.1 M sodium cacodylate for 5 minutes, dehydration was performed via an acetone series (50%, 70% and 90%; two 3 minutes washes for each concentration) to 100% (2 washes each 10 minutes). Specimens were then rinsed with 1:1 acetone:HMDS (hexamethyldisilizane) for 10 minutes, followed by two 10 minutes washes in 100% HMDS. Samples were air-dried for at least 2h before they were sputter-coated with gold.

## Competing interests

The author(s) declare that they have no competing interests.

## Authors' contributions

CM carried out the immunohistochemistry and drafted the manuscript. W-CC carried out the scanning electron microscopy. SMS and DEKF conceived of the study and helped to draft the manuscript.
